# Kawasaki-like disease and acute myocarditis in the severe acute respiratory syndrome coronavirus 2 (SARS-CoV-2) pandemic – reports of three adolescents

**DOI:** 10.17305/bjbms.2020.5037

**Published:** 2021-04

**Authors:** Vladislav Vukomanovic, Stasa Krasic, Predrag Minic, Gordana Petrovic, Dejan Nesic, Aleksandra Paripovic, Milena Vasiljevic, Borko Gobeljic

**Affiliations:** 1 Department of Cardiology, Mother and Child Health Institute of Serbia; Belgrade, Serbia; 2 School of Medicine, University of Belgrade; Belgrade, Serbia; 3 Department of Pulmonology, Mother and Child Health Institute of Serbia; Belgrade, Serbia; 4 Department of Immunology, Mother and Child Health Institute of Serbia; Belgrade, Serbia; 5 Institute of Medical Physiology “Rihard Burian”; Belgrade, Serbia; 6 Pediatric Clinic, Mother and Child Health Institute of Serbia, Belgrade, Serbia

**Keywords:** Severe acute respiratory syndrome coronavirus 2, SARS-CoV-2, myocarditis, Kawasaki disease, adolescent, coronavirus disease, COVID-19

## Abstract

The novel coronavirus disease (COVID-19) may induce multisystem inflammatory syndrome (MIS) in children, which may be associated with Kawasaki-like disease and cardiac injury. In this study, we presented three male adolescents with MIS and myocardial injury admitted to the hospital during the peak of COVID-19 pandemic. All of the three patients had a history of fever, gastrointestinal symptoms, polymorph rash, non-exudative conjunctivitis, and signs of acute myocarditis (AM). One of them had renal failure. Previously, they did not have an acute infection. Upon admission, they were hypotensive and tachycardic. A nasopharyngeal swab for severe acute respiratory syndrome coronavirus 2 (SARS-CoV-2) on reverse transcription-polymerase chain reaction (PCR) assay was negative, but neutralizing viral antibodies were positive. In combination with blood tests, electrocardiogram, echocardiography, and computerized tomography, a MIS associated with acute myocarditis with mild to moderate systolic dysfunction and dilated coronary arteries were diagnosed. Two of three patients had shock syndrome and required inotropic support. All patients were treated with intravenous immunoglobulins (Ig). The second patient had a fever up to 102.2°F (39°C) 3 days after intravenous Ig. Further, he was treated according to protocols for refractory Kawasaki disease, with an intravenous methylprednisolone pulse therapy and aspirin. After a few hours, he became afebrile and the clinical signs disappeared. The favorable short-term outcome may reflect early recognition and adequate therapy; however, the long-term outcomes are currently unknown.

## INTRODUCTION

Acute myocarditis (AM) is an inflammatory disease and cardiotropic viruses are the most commonly identified etiological factor [1], but less is known about the cardiac involvement in novel coronavirus disease (COVID-19). Increased cardiac-specific biomarkers, electrocardiographic, and echocardiographic abnormalities refer to cardiac injury due to direct, or indirect viral mechanism [2,3]. Severe acute respiratory syndrome coronavirus 2 (SARS-CoV-2) binds to cells’ viral receptors, particularly angiotensin-converting enzyme 2 (ACE2), which is expressed in the heart, which explains the link between the virus and the cardiovascular system [3]. Children had a lower number of ACE2, while the expression of the receptors in the lungs increases with age; so it could explain why children had a lower incidence and milder forms of SARS-CoV-2 infection [4]. In some children, SARS-CoV-2 sporadically (0.6-1%) inducts multisystem inflammatory syndrome (MIS) and endothelial dysfunction, with consequent multiorgan failure and clinical presentation similar to Kawasaki disease (KD) [5]. As the majority of children with MIS associated with COVID-19 MIS in children (MIS-C) have only positive viral neutralizing antibodies, MIS-C is considered a delayed complication of COVID-19, which appears most frequently 2-6 weeks after asymptomatic SARS-CoV-2 infection [6-9]. The difference between MIS-C and pediatric inflammatory multisystem syndrome associated with SARS-CoV-2 (PIMS-TS) is in anamnestic data regarding SARS-CoV-2 exposure, and positive serological or polymerase chain reaction (PCR) test [6,7].

The present report describes the three cases of MIS-C with myocardial involvement, without a history of previous signs of acute infection.

## CASE REPORT 1

**FIGURE 1:**
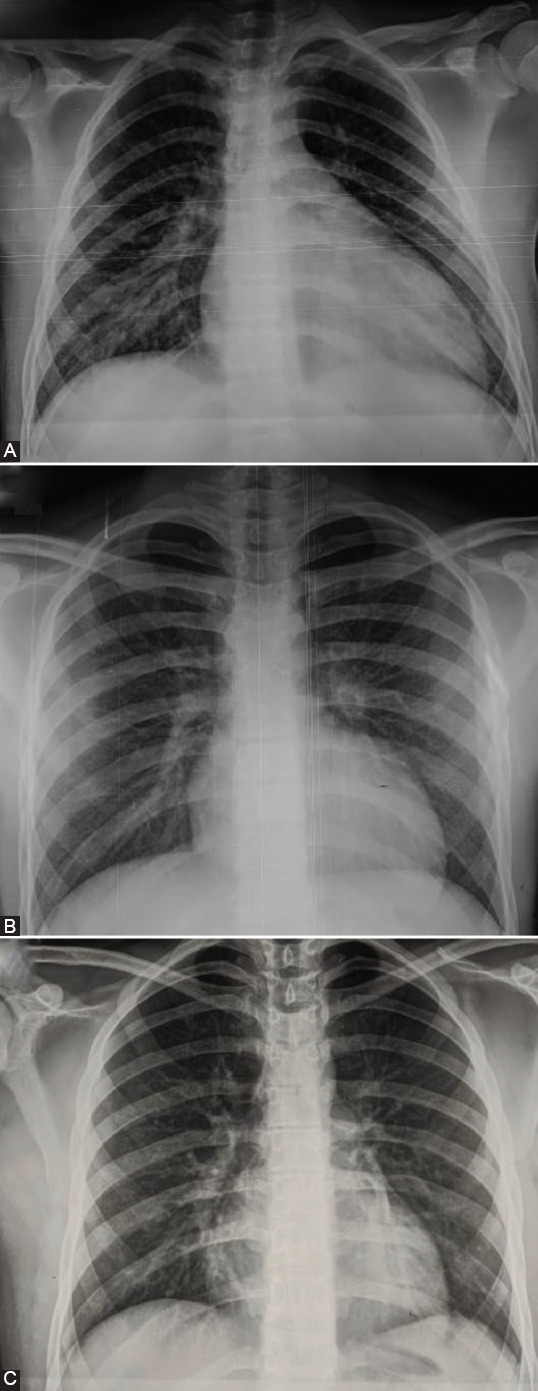
Chest radiography at the admission. (A) 1° patient: Enlarged cardiac shadow, diffusely accentuated pulmonary interstitium, bilateral pleural effusion; (B) 2° patient: Normal finding; (C) 3° patient: Diffusely accentuated pulmonary interstitium.

**FIGURE 2:**
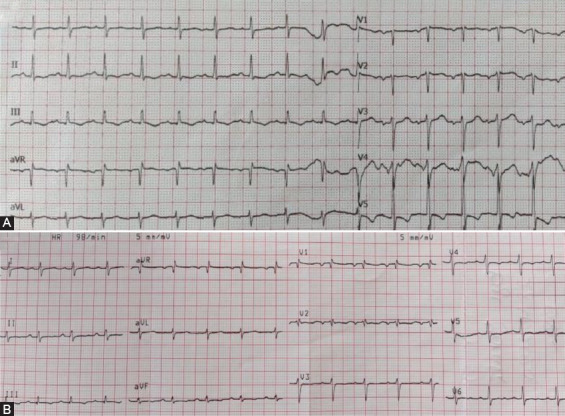
Electrocardiogram of our patients. (A) 1° patient: Negative T wave in all precordial leads; QTc interval was 0.48 s; (A) 2° patient: Negative T wave in all precordial leads QTc interval was 0.47 seconds.

A healthy 14-year-old boy was transferred to our institute due to renal failure caused by fever of 104°F, diarrhea, and vomiting for 7 previous days. Previously, he did not have signs of COVID-19. His vital parameters and laboratory analysis on admission are presented in Tables 1 and 2. Sterile pyuria was registered. X-ray and electrocardiogram (ECG) were performed (Figures 1A and 2A). Computerized tomography (CT) of the chest showed bilateral ground-glass opacities and condensations with bilateral pleural effusion. A diagnosis of suspected myocarditis was made. In a nasopharyngeal swab, SARS-CoV-2 was not detected by real-time reverse transcriptase-PCR (RT-PCR). Chromatography technique detected SARS-CoV-2-specific neutralizing antibody in the blood sample, firstly immunoglobulin G (IgG) antibody, but after 5 days, IgM and IgG were detected in paired serum samples.

**TABLE 1:**
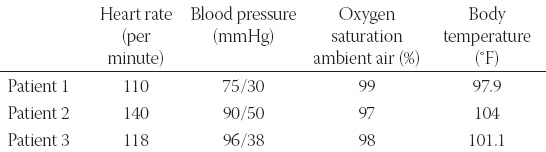
Vital parameters of our patients at the admission

**TABLE 2:**
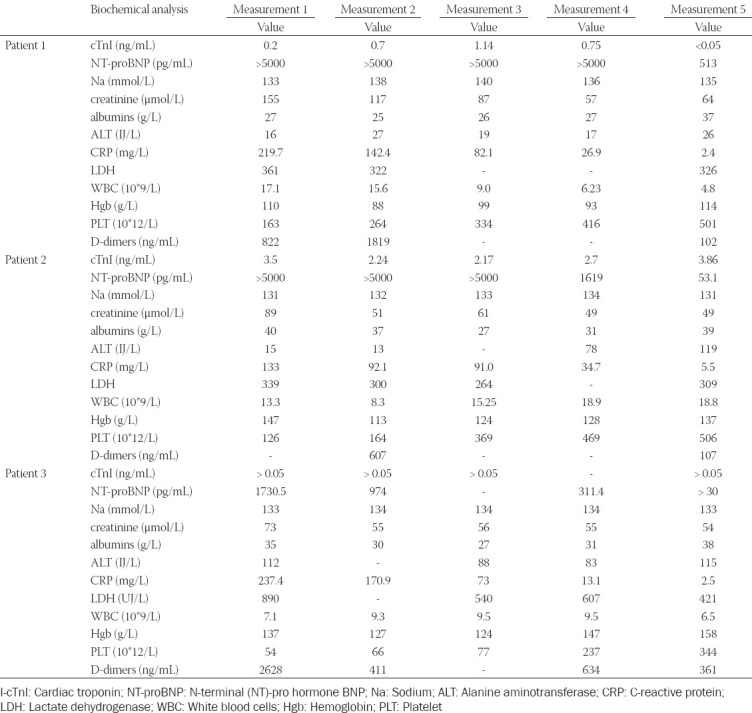
Biochemical parameters of our patient during in-hospital stay

**FIGURE 3:**
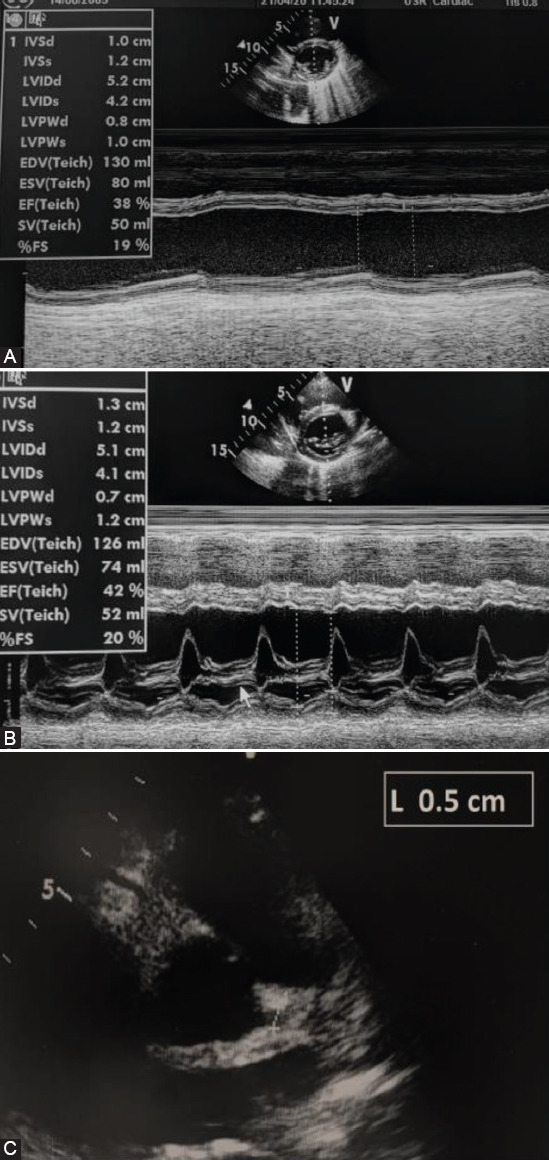
Echocardiography examination at the admission of our patients. (A) 1° patient: Left ventricular function – short axis; normal left ventricular dimensions with an increased wall thickness and decreased ejection fraction of the left ventricle. (B) 2° patient: Left ventricular function – short axis; normal left ventricular and decreased ejection fraction of the left ventricle. (C) 2° patient: Diameter of the left coronary artery (5 mm; Z score +2.77).

Transthoracic echocardiography (TTE) revealed global hypokinesis of the left ventricle (LV) with segmental hypokinesis (Figure 3A). A 9 mm pericardial effusion was notable. Antibiotic, decongestive therapy (spironolactone), and intravenous Ig (IVIG) – 2 g/kg/48 h were administered. Fraxiparine was administered in the prophylactic dose. The patient remained hypotensive, oliguric and required inotropic support. Blood pressure progressively stabilized and dopamine was weaned on day 2. During hospitalization, the patient had a polymorphic rash and palmar erythema. He became afebrile on day 3. TTE on discharge showed normalization of systolic function (left ventricular ejection fraction [LVEF] 68%).

Cardiovascular magnetic resonance (CMR) examination was performed 2 weeks after discharge, and it was shown mild decreased LVEF.

## CASE REPORT 2

A healthy 14-year-old boy was admitted due to fever, vomiting, and headache for 7 previous days. His medical history did not reveal signs of acute infection. His vital parameters and laboratory analysis on admission were presented in Tables 1 and 2. The first ECG record was normal, but during hospitalization, ECG showed inversion of T wave in all precordial leads with prolonged QTc interval (Figure 2B). X-ray finding was normal (Figure 1B). A diagnosis of suspected myocarditis was made. A nasopharyngeal swab was negative for SARS-CoV-2. SARS-CoV-2-specific neutralizing IgG antibody was detected firstly, and IgM and IgG were detected in paired serum samples.

TTE revealed hypokinesis of LV (Figure 3B) and dilated left main coronary artery (LCA) (Figure 3C). Pericardial effusion was 7 mm. He was treated with antibiotics, decongestive therapy (spironolactone), and IVIG. He remained hypotensive, oliguric and required inotropic drug support. Dopamine treatment was weaned on day 3. Fraxiparine was administered in the prophylactic dose and replaced by aspirin after 10 days. Despite the treatment, he had a fever of 102.2°F 3 days after IVIG. Bilateral bulbar non-exudative conjunctivitis and polymorphic rash were developed on the 3^rd^ day of in hospital treatment. We concluded that our patient had atypical IVIG refractory KD. An intravenous methylprednisolone pulse (IVMP) was administered. After the first IVMP, the clinical signs disappeared. LVEF and LCA became normal (LVEF 63%, LCA 3.5 mm). On discharge, the skin from his hands was peeling off.

CMR examination was performed 5 weeks after the onset of the disease, and it was shown a normal ventricles function and morphology of the LCA.

## CASE REPORT 3

A healthy 17-year-old boy was transferred to our institute due to suspicion of MIS-C with myocardial injury. The disease started with a fever up to 104F, headache, malaise, diarrhea, and dyspnea 5 days before admission. The patient had no previous signs of acute infection. Laboratory analysis before the transfer showed elevated cTnI value. He had anamnestic data about contacts with SARS-CoV-2 positive persons. On admission, he was febrile, hypotensive, and tachycardic (Table 1). He had a polymorphic rash, palmar erythema, and bilateral bulbar non-exudative conjunctivitis and suffusions. His laboratory analysis on admission is presented in Table 2. ECG on admission was normal, but during the in-hospital stay, prolonged QTc was observed (QTc 0.47 seconds). X-ray was performed (Figure 1C). A chest CT showed sporadic bilateral pulmonary fibrosis. In a nasopharyngeal swab, SARS-CoV-2 was not detected by RT-PCR. SARS-CoV-2-specific neutralizing IgG antibody in the blood sample was detected by enzyme-immuno-essay technique (ELISA).

TTE revealed mild hypokinesis of LV (LVEF 55%) and dilated coronary arteries (LCA 5 mm, Z score +2.14; right CA 4-5 mm, Z score +2.5). The patient was treated with antibiotics, corticosteroids (dexamethasone), and IVIG. Dopamine treatment was weaned off after 24 hours. He became afebrile on day 2. TTE after IVIG treatment showed normalization of systolic function (LVEF 68%) and diameter of coronary arteries (LCA, RCA 3 mm). Fraxiparine was replaced by aspirin after 5 days. He was discharged from hospital on day 12.

## DISCUSSION

Our report presents three male adolescents who were admitted to our hospital within 2 months from the beginning and at the height of the SARS-CoV-2 epidemic in Serbia, with similar clinical presentation and clinical course. All of them had a history of high fever, gastrointestinal symptoms, but on admission, we observed polymorph rash, palmar erythema, and signs of AM. In addition, two of them also had bilateral bulbar non-exudative conjunctivitis. Consequently, their clinical presentation, in combination with echocardiography, and laboratory parameters, as well as positive serology test for SARS-CoV-2, point to MIS-C with simultaneous cardiac involvement. The appearance of SARS-CoV-2-neutralizing antibodies in patients’ serum is more common than viral nucleic acid isolation by RT-PCR, while MIS-C might be a postponed complication of COVID-19 [5-9]. Verdoni et al. showed that the incidence of KD-like increased 30-fold in the past months, and one-half of patients had incomplete KD. According to literature data, patients with MIS-C were older in comparison to patients with classic KD [5-9], as well as in our cases. Patients with KD might develop severe LV dysfunction with consequent hypotension and malperfusion syndrome, which is known as KD shock syndrome (KDSS) [5]. The prevalence of KDSS was 2-7% before SARS-CoV-2 pandemic, but during the pandemic, more than one half (in some studies to 82%) of patients had KDSS [5,6,8]. Two of our patients with shock syndrome required cardiac inotropic support due to myocardial injury with moderate LV systolic dysfunction and all of them were treated with dopamine due to hypotension and oliguria. The patients were treated with IVIG according to the protocol for KD and AM treatment. Two adolescents became afebrile in the 1^st^ hours of IVIG treatment, but one stayed febrile 3 days after IVIG-administration. As his clinical presentation was very similar to refractory KD, he was treated according to protocols for this condition. After a few hours of IVMP administration, our patient became afebrile, and clinical signs of MIS-C disappeared. Patients with MIS-C had a more pronounced inflammation; severe disease course was refractory to IVIG treatment and with consequent more common steroid use than patients with classic KD [5], as well as in our case series.

Two-thirds of patients with MIS-C had cardiac involvement and some of them required inotropic support which is not common in classic KD [5-9]. Cardiac and conduction system injury include echocardiography abnormalities, elevated levels of cardiac-specific markers, blood pressure abnormalities, arrhythmias and repolarization aberrations [2,5]. In our case series, all patients had prolonged QTc interval during the in-hospital stay, increased level of cardiospecific markers, and mild to moderate systolic dysfunction evaluated by echocardiography examination. According to Matsubara et al., patients with MIS-C had lower LV ejection fraction and Fractional shortening compared to children with classic KD. In addition, MIS-C patients had more common mitral valve regurgitation, pericardial, and pleural effusion. On the other hand, children with classic KD had more frequent coronary artery (CA) aneurysm [9]. Ectasias of CAs were observed on admission in two of our patients, but on discharge, CAs were of appropriate diameter.

The etiopathogenesis of cardiac involvement in MIS-C may reflect a process of direct cardiomyocytes damage, due to viral acting on ACE2 receptors, with their consequent downregulation and homeostasis disruption [1,2]. The most recent autopsy of the heart material of an 11-year-old child with fulminant AM showed viral particles in the heart muscle, while previous autopsies of SARS-CoV-2 positive patients showed inflammatory infiltrates composed of macrophages and sporadic CD4+ T cells with no sign of lymphocytic myocarditis [10]. Indirect injury of the cardiovascular system was explained by SARS-CoV-2 potential to induce macrophage activation, cytokines storm, and secondary hemophagocytic lymphohistiocytosis with consequent multiorgan injury [1-3,5]. Myocardial damage might also be a consequence of microvascular occlusion by microthrombi or direct endothelial or vascular injury, as well as hypoxemia due to respiratory tract injury [6-9]. Taken together, the mechanism of cardiomyocyte damage during MIS-C remains unknown.

On discharge, our patients had normal echocardiography and CMR findings. During a short-term period, most of the MIS-C patients recovered systolic function, but diastolic dysfunction persisted [9].

Our patients had a similar clinical presentation, laboratory, and echocardiography findings, which point to MIS-C with a cardiac injury. Two of them had KDSS and one had refractory KD with a prompt response to IVMP therapy. Some elements of the disease that occurred in the COVID-19 pandemic point to MIS with a consequential cardiac injury similar to KD and KDSS. The course of the disease and the effect of immunomodulatory therapy may indicate a disturbed immune response to a currently unknown agent, which might be SARS-CoV-2.
